# Various Terpenoids Derived from Herbal and Dietary Plants Function as PPAR Modulators and Regulate Carbohydrate and Lipid Metabolism

**DOI:** 10.1155/2010/483958

**Published:** 2010-06-03

**Authors:** Tsuyoshi Goto, Nobuyuki Takahashi, Shizuka Hirai, Teruo Kawada

**Affiliations:** ^1^Department of Applied Life Science, Faculty of Applied Biological Sciences, Gifu University, Gifu 501-1193, Japan; ^2^Laboratory of Molecular Function of Food, Division of Food Science and Biotechnology, Graduate School of Agriculture, Kyoto University, Uji 611-0011, Japan; ^3^Laboratory of Food Nutrition, Division of Applied Biological Chemistry, Graduate School of Horticulture, Chiba University, 648 Matsudo, Chiba 271-8510, Japan

## Abstract

Several herbal plants improve medical conditions. Such plants contain many bioactive phytochemicals. Terpenoids (also called “isoprenoids”) constitute one of the largest families of natural products accounting for more than 40,000 individual compounds of both primary and secondary metabolisms. In particular, terpenoids are contained in many herbal plants, and several terpenoids have been shown to be available for pharmaceutical applications, for example, artemisinin and taxol as malaria and cancer medicines, respectively. Various terpenoids are contained in many plants for not only herbal use but also dietary use. In this paper, we describe several bioactive terpenoids contained in herbal or dietary plants, which can modulate the activities of ligand-dependent transcription factors, namely, peroxisome proliferator-activated receptors (PPARs). Because PPARs are dietary lipid sensors that control energy homeostasis, daily eating of these terpenoids might be useful for the management for obesity-induced metabolic disorders, such as type 2 diabetes, hyperlipidemia, insulin resistance, and cardiovascular diseases.

## 1. Diversity of Terpenoids in Nature

Nature relies on an intricate network of biosynthetic pathways to produce a lot of small organic molecules required to support life. Terpenoids (also called “isoprenoids”) constitute one of the largest families of natural products accounting for more than 40,000 individual compounds of both primary and secondary metabolisms. Most of them are of plant origin, and hundreds of new structures are reported every year [1–3]. All organisms naturally produce some terpenoids as part of primary metabolism, but many produce terpenoids via secondary metabolism.

**Figure 1:**
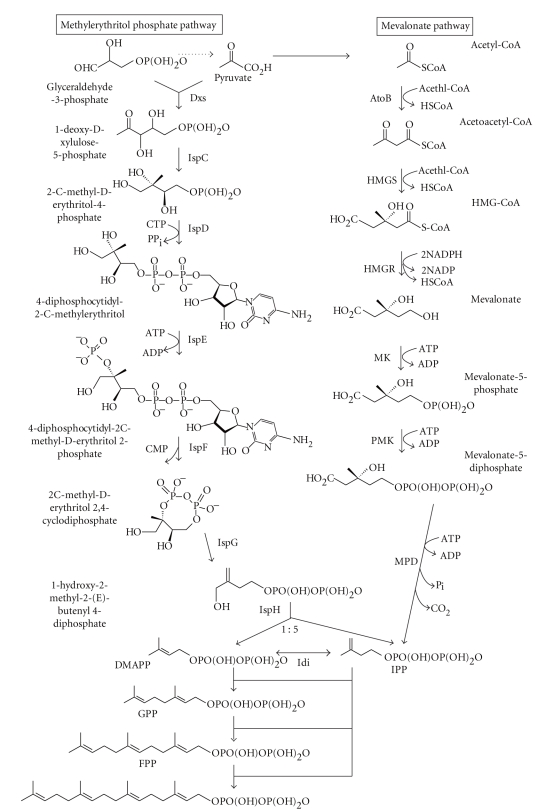
Biosynthetic routes to polyprenyl pyrophosphate terpenoid biosynthetic pathways. Dxs: 1-deoxy-d-xylulose-5-phosphate synthase; IspC: 1-deoxy-d-xylulose-5-phosphate reductoisomerase; IspD: 4-diphosphocytidyl-2-C-methyl-d-erythritol synthase; IspE: 4-diphosphocytidyl-2-C-methyl-d-erythritol kinase; IspF: 2-C-methyl-d-erythritol2,4-cyclodiphosphate synthase; IspG: 1-hydroxy-2-methyl-2-(E)-butenyl 4-diphosphate synthase; IspH: 1-hydroxy-2-methyl-2-(E)-butenyl 4-diphosphate reductase; AtoB, acetyl-CoA C-acetyltransferase; HMGS: hydroxymethylglutaryl-CoA synthase; HMGR: hydroxymethylglutaryl-CoA reductase; MK: mevalonate kinase; PMK: phosphomevalonate kinase; MPD: mevalonate pyrophosphate decarboxylase; Idi: isopentenyl pyrophosphate isomerase; GPP: geranyl pyrophosphate; FPP: farnesyl pyrophosphate; GGPP: geranylgeranyl pyrophosphate.

Isopentyl diphosphate (IPP) and its isomer dimethylallyl diphosphate (DMAPP) are the universal five-carbon precursors of all terpenoids. After the discovery of the mevalonate (MVA) pathway in yeast and animals, it was assumed that IPP was synthesized from acetyl-CoA via MVA and then isomerized to DMAPP in all eukaryotes and some Gram-positive prokaryotes [2, 3] (Figure 1). The origins of archeal terpenoids were unknown until recently, when four of the six enzymes have been identified to be present in sequenced genomes [2]. Recently, a nonstandard MVA pathway involving phosphorylation of isopentenyl phosphate has been discovered in *Methanocaldococcus jannaschii *[4]. Before 1993, the MVA pathway was the only known source of terpenoids. After isotope-labeling studies by Rohmer et al. [5], it has been shown that there is an alternate pathway to terpenoids that do not originate from acetyl-CoA. The complete pathway has been finally elucidated in 2002 [6]. This alternative MVA-independent pathway has been named the methylerythritol phosphate (MEP) pathway (Figure 1), which has been identified in both bacteria and plants [2, 3]. Plants use both pathways although they are compartmentalized: MVA to the cytoplasm and possibly to mitochondria to provide sterols, the side chain of ubiquinone, and sesquiterpenes (C15), and MEP to plastids providing plastidial terpenoids, for example, isoprene (C5), monoterpenes (C10), diterpenes (C20, including gibberellins and the phytyl tail of tocopherols and chlorophylls), and carotenoids (C40) [3]. Moreover, there is evidence that a certain degree of crosstalk between the MVA and MEP pathways can occur, implying that these pathways are not completely autonomous [7].

Plants have an enormous capacity to synthesize huge amounts of diverse terpenoids, particularly via the combination of the terpenoid biosynthetic route and other secondary metabolic pathways. For instance, tocopherol biosynthesis occurs as a result of combination of the shikimate and isoprenoid pathways, which lead to homogentisic acid phytyl diphosphate, which in combination ultimately lead to the formation of tocopherols (formed by a chromanol head group and a phytyl tail) [8].

In addition to universal physiological, metabolic, and structural functions, many specific terpenoids function in various situations, including communication and defense. Members of the isoprenoid group also include industrially useful polymers (e.g., rubber and chicle) and agrochemicals (e.g., pyrethrins and azadirachtin).

It is known that several herbal plants improve medical conditions. Such plants contain many bioactive phytochemicals. In particular, terpenoids are contained in many herbal plants, and several terpenoids have been shown to be available for pharmaceutical applications, for example, artemisinin and taxol as malaria and cancer medicines, respectively. Various terpenoids are contained in many plants for not only herbal medicine use but also dietary use [9].

**Figure 2:**
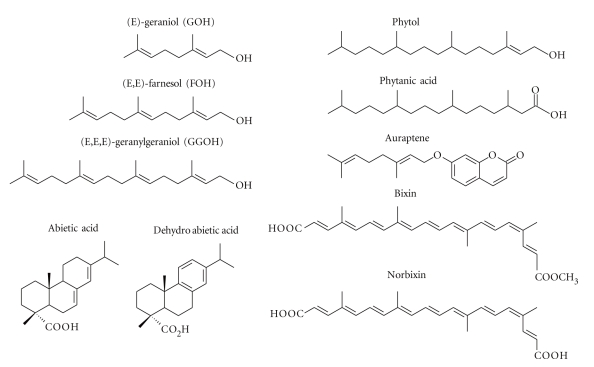
Chemical structures described in this paper.

In this paper, we describe several bioactive terpenoids (Figure 2) contained in herbal or dietary plants, which have the potential to ameliorate metabolic disorders via activation of ligand-dependent transcription factors, namely, peroxisome proliferator-activated receptors (PPARs).

## 2. PPARs: Therapeutic Targets of Metabolic Syndrome

### 2.1. Nuclear Receptors

Members of the nuclear receptor superfamily of ligand-dependent transcription factors play a multitude of essential roles in development, homeostasis, reproduction, and immune functions [10–14]. Ligand binding induces a conformational change in nuclear receptors, releasing corepressors in exchange for coactivators. Ligand-activated complexes recruit basal transcriptional machineries, resulting in an enhanced gene expression. Nuclear receptors include the classical steroid hormone receptors (estrogen, progesterone, androgen, glucocorticoid, and mineralcorticoid receptors); “orphan receptors,” which exhibit conserved features of the nuclear receptor family but have not been linked to endogenous ligands; and so-called “adopted orphan receptors,” which were initially identified as orphan receptors but were subsequently linked to endogenous ligands. The adopted orphan receptors include the thyroid hormone receptors, retinoic acid receptors, PPARs, and liver X receptors (LXRs). Nearly all members of this family contain a highly conserved DNA-binding domain that mediates sequence-specific recognition of target genes and a C-terminal domain that determines the specific ligand-binding properties of each receptor and mediates ligand-regulated transcriptional activation and/or repression [10].

Nuclear receptors are frequent biological targets of active compounds contained in herbal and dietary plants. This is perhaps not surprising, since nuclear receptors evolved to be regulated by lipophilic molecules derived from diet and the environment [15, 16]. At least ten of these receptors have been shown to be directly activated by compounds purified from herbal remedies [16]. Some compounds have a complex pharmacology; for example, grapeseed-derived resveratrol is a ligand of estrogen receptors and PPARs [17, 18] but has also been suggested to activate sirtuin 1 (SIRT1), an NAD^+^-dependent protein deacetylase enzyme implicated in the biology of aging [19]. Other phytochemicals target multiple nuclear receptors [16].

### 2.2. PPARs

PPARs are members of the nuclear receptor superfamily, which are activated by fatty acids and their derivatives. PPARs are dietary lipid sensors that regulate lipid and carbohydrate metabolism [20]. In mammals, three subtypes of PPAR, *α*, *δ*, and *γ*, were found [21]. PPARs form heterodimers with retinoid-X-receptors (RXRs) and bind to consensus DNA sites composed of direct repeats (DRs) of hexameric DNA sequences (AGGNCA) separated by 1 bp (DR1). In the absence of ligands, PPAR-RXR heterodimers recruit corepressors and associated histone deacetylases and chromatin-modifying enzymes, silencing transcription by so-called active repression [22–24]. Ligand-binding induces a conformational change in PPAR-RXR complexes, releasing repressors in exchange for coactivators. Ligand-activated complexes recruit the basal transcriptional machinery, resulting in an enhanced gene expression.

PPAR*α* is highly expressed in the liver, cardiac muscle, and digestive tract, and regulate the expression of target genes involved in lipid catabolism. Activators of PPAR*α*, such as fibrates, decrease circulating lipid levels and are commonly used to treat hypertriglyceridemia and other dyslipidemic states [25]. PPAR*δ* is expressed in many tissues including the skeletal muscle and brown adipose tissue. Recently, it has been suggested that PPAR*δ* activation attenuates obesity and type-2 diabetes [26, 27]. PPAR*γ* is abundant in adipose tissues functioning as the key transcription factor for adipogenesis. Synthetic ligands for PPAR*γ*, such as thiazolidinediones, are increasingly used to treat type-2 diabetes [28].

PPARs are involved in not only energy homeostasis but also inflammation. PPAR*α* regulates inflammatory processes, mainly by inhibiting inflammatory gene expression. In recent years, several molecular mechanisms responsible for the immunosuppressive effects of PPAR*α* have been uncovered [29]. These include interference with several proinflammatory transcription factors [30]. The number of studies that have addressed the role of PPAR*δ* during inflammation is limited. So far, an anti-inflammatory effect has been observed in macrophages suggesting a possible role for PPAR*δ* in the process of atherogenic inflammation. [31]. Similar to PPAR*α*, PPAR*γ* is involved in governing inflammatory response, particularly in macrophages. Currently, two different molecular mechanisms have been proposed by which anti-inflammatory actions of PPAR*γ* are effectuated: (1) via interference with proinflammatory transcription factors [32] and (2) by preventing removal of correpressor complexes from gene promoter regions resulting in suppression of inflammatory gene transcription [33].

Recently, it has been indicated that obesity is associated with a low-grade chronic inflammation state [34]. The inflammatory condition in obesity is increasingly being recognized as an important contributor to the development of metabolic syndrome and its associated complications. Adipocytes can secret cytokines involved in inflammation, such as adiponectin, monocyte chemoattractant protein-1 (MCP-1), and tumor necrosis factor-*α* (TNF-*α*) [35]. MCP-1, a member of the CC chemokine superfamily, plays a pivotal role in monocyte/macrophage trafficking and activation [36]. Macrophages also produce various proinflammatory factors including MCP-1 and TNF-*α*. Macrophage-derived TNF-*α* establishes a vicious cycle that augments inflammatory changes and insulin resistance in obese adipose tissues [37]. Therefore, to prevent obesity-related inflammation, it is important to decrease the production of obese-adipose-tissue-derived proinflammatory factors such as MCP-1 and TNF-*α*.

Several herbal and dietary plants improve medical conditions including diabetes mellitus, hyperlipidemia, and cardiovascular disease associated with an abnormality of lipid metabolism [38, 39]. To screen for novel natural ligands for PPARs, we have evaluated PPAR ligand activities for various terpenoids in an advanced highly sensitive system with the coexpression of a coactivator for nuclear receptors, the cAMP-response element-binding protein (CREB)-binding protein (CBP), developed by modifying the luciferase reporter assay system [40]. Hereinafter, we describe several terpenoids, identified as novel PPAR ligands, in our PPAR ligand screening.

## 3. Novel Functions of Dietary Terpenoidsas PPAR Ligands

### 3.1. Isoprenols

We carried out screening for novel PPAR ligands in natural compounds contained in medicinal plants. We used several terpenoids including carotenoids and polyisoprenoid alcohols (isoprenols) for the screening, because these compounds are contained in many medicinal and dietary plants [41]. These terpenoids have multifunctions such as the suppression of tumor proliferation [41–43], apoptosis-inducing activity [9], and cation channel regulation [44]. Some terpenoids, which are intermediates in cholesterol synthesis, regulate the activity of 3-hydroxy-3-methylglutaryl-coemzyme A (HMG-CoA) reductase, a key enzyme in cholesterol synthesis by controlling the degradation of the enzyme [45, 46]. Such functions of dietary terpenoids are significant for the trials to manage disease conditions such as cancers or cardiovascular diseases using food factors.

**Figure 3:**
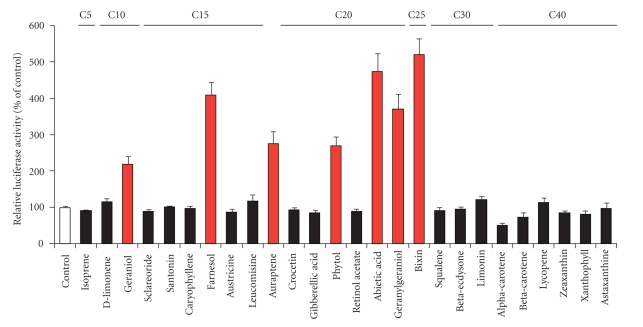
Effects of various terpenoids on the activation of PPAR*γ* ligand and chemical structures of active terpenoids. PPAR*γ* activity in monkey CV-1 kidney cells was determined by luciferase reporter assay using advanced highly sensitive GAL4/PPAR*γ* chimera system described in [40]. After 24 hours of incubation with or without each terpenoid at 50 or 100 *μ*M, luciferase activity was measured. The activity of a vehicle control was set at 100% and the relative luciferase activities are presented as fold induction compared with that of the vehicle control. The values are means ± S.E.M. of 3-4 replicates. B: Chemical structures of active terpenoids.

As shown in Figure 3, several terpenoids activated PPAR*γ* at a concentration of 50 or 100 *μ*M [40]. In this assay, we identified that isoprenols, such as geraniol, farnesol, and geranylgeraniol (chemical structures are shown in Figure 2) have a potential to activate PPAR*γ* as novel ligands. At 100 *μ*M, the activations by geraniol, farnesol, and geranylgeraniol were 2.2-, 4.1-, and 3.7-fold that by the vehicle control, respectively. On the other hand, squalene had no effect on PPAR*γ* transactivation. Although geraniol had no effect on PPAR*α*, farnesol and geranylgeraniol also dose-dependently activated PPAR*α* (10- and 8.6-fold increases at a concentration of 100 *μ*M compared with vehicle controls, respectively) in the PPAR*α* ligand assay system [40]. These activities were so potent and nearly the same as that of 10 *μ*M fenofibrates, one of the fibrates (antihyperlipidemia drugs) used as a positive control for PPAR*α*. In this regard, these farnesol and geranylgeraniol isoprenols have the effects of dual activation of PPAR*γ* and PPAR*α*.

PPAR*γ* activation in adipose tissues results in the improvement of insulin resistance [46] and PPAR*α* activation in the liver induces the lowering of circulating lipid levels [47]. These effects are due to the regulation at mRNA expression levels of target genes of PPARs. The addition of each isoprenol induced mRNA expression of PPAR target genes in 3T3-L1 adipocytes and HepG2 hepatocytes [40]. Therefore, it is possible that isoprenols could regulate insulin resistance and/or circulating lipid levels. The finding of the dual activation of PPAR*γ* and PPAR*α* by isoprenols is very important for thinking of the mechanisms understanding the effects of medicinal plants and valuable for the management of diabetic and hyperlipidemic conditions in herbal medicine. Indeed, in our preliminary study, farnesol ameliorated hyperglycemia in high fat diet-fed wild-type mice but not in PPAR*α* deficient mice (Goto et al., unpublished data). These findings indicate that improvement of obesity-associated metabolic disorders by farnesol is mainly dependent on PPAR*α* activation. These results provide not only a significant molecular basis on how herbal plants containing phytochemicals such as isoprenols induce the improvement of diabetes or hyperlipidemia, but also possibilities that phytochemicals might have therapeutic applications in lipid abnormalities, such as obesity, diabetes mellitus, and hyperlipidemia.

### 3.2. Phytol

Phytol, a diterpene alcohol, which is a carbon side chain of a chlorophyll molecule (Figure 2), is involved in the production of energy from light. Phytol is a plastidial terpenoid and synthesized via the MEP pathway in plastids [48]. Since almost all photosynthetic organisms use chlorophylls, phytol is also abundantly present in nature including various vegetables. It is suggested that chlorophyll molecules are partially digested and the phytol moiety is released in animals [49]. Then, the released phytol is absorbed in the small intestine and converted to phytanic acid in the liver.

Phytanic acid is a branched-chain, terpenoid-derived fatty acid constituent of diet (Figure 2). In surveys of phytanic acid content of a variety of food products, high levels were indeed found in products such as milk, butter, cheese, meat from cows, sheep, and some fish and fish oils, whereas no phytanic acid is present in vegetables [50]. This compound can also be produced from the conversion of dietary phytol in the body [51]. Phytanic acid has been reported to activate PPAR*γ* and the retinoid-X-receptor (RXR) [52, 53] so that differentiation is stimulated in both white and brown adipocytes [52]. In addition, phytanic acid stimulates PPAR*α* to regulate lipid metabolism in some types of cell [54]. Therefore, the intake of phytol as a precursor of phytanic acid may be valuable for the management of lipid metabolism through the activation of PPARs. Indeed, a phytol-enriched diet increased plasma and hepatic levels of phytanic acid, and induced the mRNA expression of PPAR*α* target genes involved in peroxisomal and mitochondrial *β*-oxidation and fatty acid metabolism [55].

However, since the conversion of phytol into phytanic acid is not very rapid, a phytol-enriched diet also induced accumulation of phytol in the liver [56]. Moreover, as described previously, several terpenoids, which resemble phytol in structures, activate PPARs in adipocytes and hepatocytes [40]. In this sense, it must be valuable to analyze the effects of phytol itself as an activator of PPARs.

Therefore, we evaluated the effects of phytol on PPAR activity using our advanced highly sensitive luciferase assays (Figure 3). Among the PPAR isoforms, PPAR*α* was activated the most markedly following the addition of phytol [57]. The effects of phytol on PPAR*α* activation were larger than those of phytanic acid under our experimental conditions. Phytol induced the mRNA and protein expression of PPAR target genes in a manner dependent on the level of PPAR*α* expression in HepG2 hepatocytes. In our in vitro coactivator recruiting assay, it was revealed that phytol can activate PPAR*α* directly [57]. These findings indicate that phytol itself can directly bind to PPAR*α* as its ligand.

Because the activation of PPAR*α* is one of the most important factors in lipid metabolism in peripheral tissues including the liver and muscles, the ability of phytanic acid and phytol to activate PPAR*α* is very important in the management of lipid metabolism using food factors. Induction of PPAR*α* target gene expression in mice fed a phytol-enriched diet [55] is likely due to not only phytanic acid but also phytol. It is considered that such effects of phytol are valuable for the control of lipid abnormalities in common diseases including obesity, diabetes, and hyperlipidemia through PPAR*α* activation in the liver.

### 3.3. Abietic Acid Derivatives

The amount of variety of hydrocarbons and their derivatives used in industrial and commercial activities has been increasing over the years. Abietic acid is a tricyclic-diterpene carboxylic acid (Figure 2), and is the main component of the rosin fraction of oleoresin synthesized by conifer species, such as grand fir (*Abies grandis*) and lodgepole pine (*Pinus contorta*) [58]. Abietic acid is commonly used as a fluxing agent in solder, as a paper sizing agent to make paper more water resistant, and in printing inks, adhesives, and plasticizers [59]. Moreover, it has been reported that abietic acid is a bioactive compound and it has an anti-inflammatory effect. In lipopolysaccharide (LPS)-stimulated macrophages, abietic acid suppresses production of prostaglandin E2 (PGE2) in vitro and in vivo [60].

To investigate whether the activation of PPARs is related to the anti-inflammatory effects of abietic acid and its derivatives, we evaluated the effects of abietic acid and its derivatives on PPAR activity (Figure 2). Abietic acid and dehydroabietic acid, one of major components of colophony (also known as Rosin and pine resin), potently activated both PPAR*α* and PPAR*γ* but not PPAR*δ* [61, 62]. Similarly to thiazolidinedione, a synthetic PPAR*γ* ligand, abietic acid suppressed mRNA expressions of TNF-*α* and cyclooxygenase 2 (COX2), which are induced in inflammatory reactions, in LPS-stimulated macrophages [61]. Dehydroabietic acid stimulated PPAR*α* and PPAR*γ* more potently than abietic acid [62]. Dehydroabietic acid significantly suppressed the production of proinflammatory mediators such as MCP-1, TNF-*α*, and NO in LPS-stimulated macrophages and in the coculture of macrophages and adipocytes [62]. In obese diabetic KK-Ay mice, dietary dehydroabietic acid suppressed obesity-associated elevation of circular MCP-1 and TNF-*α* levels and their mRNA expressions in white adipose tissues. Moreover, dehydroabietic acid improved carbohydrate and lipid metabolism [63]. These findings indicate that the anti-inflammatory effects of abietic acid and dehydroabietic acid are at least partly due to the activation of PPARs. Additionally, it is suggested that these compounds can be used not only for anti-inflammation but also for regulating carbohydrate and lipid metabolism and atherosclerosis.

### 3.4. Auraptene

Citrus-fruit-derived compounds have many beneficial bioactivities (e.g., anticarcinogenic, antihypertension, and anticardiovascular disease effects) [64, 65]. Through our screening for PPARs ligands (Figure 2), we identified auraptene, a geraniol coumarin ether, as a novel PPAR*α* and PPAR*γ* ligand [66, 67]. Auraptene (Figure 2) occurs in a variety of citrus fruits. It has been reported that auraptene has anti-inflammatory and anticarcinogenic activities. In cultured adipocytes, auraptene upregulated an antiatherosclerotic, antidiabetic, and anti-inflammatory cytokine, adiponectin, and downregulated a proinflammatory cytokine, MCP-1. These effects disappeared in the presence of GW9662, a PPAR*γ* antagonist [66], suggesting that auraptene improves adipocytokine profiles via the activation of PPAR*γ*. In addition, mRNA expressions of several PPAR*α* target genes involved in FA catabolism, were also induced in PPAR*α*-expressing HepG2 hepatocytes by auraptene treatment [67]. It is likely that auraptene regulates the mRNA expressions of both PPAR*γ* and PPAR*α* target genes as a dual agonist, and these activities might contribute to the anti-cardiovascular disease effect of citrus fruits.

### 3.5. Bixins

Annatto obtained from the pericarp of seeds from *Bixa orellana* is a natural pigment extensively used in many processed foods [68, 69]. Bixin and norbixin (Figure 2), which are carotenoids, are the main components of this pigment, and have been reported to possess antioxidative and anticarcinogenic effects [70–72]. Furthermore, it has also been indicated that both annatto extract and norbixin have hypoglycemic effects in nonobese dogs and mice, respectively [73, 74]. In PPAR*γ* ligand assay, both bixin and norbixin activated PPAR*γ* (Figure 2), and bixin induced PPAR*γ* target genes in 3T3-L1 adipocytes, resulting in the promotion of adipocyte differentiation and insulin-stimulated glucose uptake [75]. Therefore, the hypoglycemic effects of annatto and norbixin might be caused by the activation of PPAR*γ*.

## 4. Conclusions

In this paper, we mentioned the diversity of terpenoids, functions of PPARs, and several terpenoids activating PPARs. The prevalence of obesity worldwide has progressively increased over the past decades. This unabated rise has spawned proportionate increases in obesity-associated metabolic disorders. Currently, synthetic PPAR agonists are widely used for the treatment of metabolic disorders. Daily intake of dietary terpenoids, which activate PPARs as we described above, may be valuable for the control of carbohydrate and lipid disorders. Dietary patterns rich in vegetables and fruit are associated with a lower prevalence of metabolic syndrome [65, 76]. Because most of the terpenoids are of plant origin and they are contained in vegetables and fruit, dietary terpenoids may contribute to a decrease in risk of metabolic syndrome. Moreover, because the terpenoids constitute one of the largest families of natural products, more potent and useful PPAR activators may exist.

## References

[1] Sacchettini JC, Poulter CD (1997). Creating isoprenoid diversity. *Science*.

[2] Withers ST, Keasling JD (2007). Biosynthesis and engineering of isoprenoid small molecules. *Applied Microbiology and Biotechnology*.

[3] Penuelas J, Munne-Bosch S (2005). Isoprenoids: an evolutionary pool for photoprotection. *Trends in Plant Science*.

[4] Grochowski LL, Xu H, White RH (2006). Methanocaldococcus jannaschii uses a modified mevalonate pathway for biosynthesis of isopentenyl diphosphate. *Journal of Bacteriology*.

[5] Rohmer M, Knani M, Simonin P, Sutter B, Sahm H (1993). Isoprenoid biosynthesis in bacteria: a novel pathway for the early steps leading to isopentenyl diphosphate. *Biochemical Journal*.

[6] Rohdich F, Hecht S, Gärtner K (2002). Studies on the nonmevalonate terpene biosynthetic pathway: metabolic role of IspH (LytB) protein. *Proceedings of the National Academy of Sciences of the United States of America*.

[7] Laule O, Furholz A, Chang H-S (2003). Crosstalk between cytosolic and plastidial pathways of isoprenoid biosynthesis in Arabidopsis thaliana. *Proceedings of the National Academy of Sciences of the United States of America*.

[8] Munné-Bosch S, Alegre L (2002). The function of tocopherols and tocotrienols in plants. *Critical Reviews in Plant Sciences*.

[9] Mo H, Elson CE (1999). Apoptosis and cell-cycle arrest in human and murine tumor cells are initiated by isoprenoids. *Journal of Nutrition*.

[10] Evans RM (1988). The steroid and thyroid hormone receptor superfamily. *Science*.

[11] Mangelsdorf DJ, Thummel C, Beato M (1995). The nuclear receptor super-family: the second decade. *Cell*.

[12] Mangelsdorf DJ, Evans RM (1995). The RXR heterodimers and orphan receptors. *Cell*.

[13] Giguere V (1999). Orphan nuclear receptors: from gene to function. *Endocrine Reviews*.

[14] Glass CK (2006). Going nuclear in metabolic and cardiovascular disease. *Journal of Clinical Investigation*.

[15] Chawta A, Repa JJ, Evans RM, Mangelsdorf DJ (2001). Nuclear receptors and lipid physiology: opening the X-files. *Science*.

[16] Lazar MA (2004). East meets West: an herbal tea finds a receptor. *Journal of Clinical Investigation*.

[17] Klinge CM, Risinger KE, Watts MB, Beck V, Eder R, Jungbauer A (2003). Estrogenic activity in white and red wine extracts. *Journal of Agricultural and Food Chemistry*.

[18] Inoue H, Jiang X-F, Katayama T, Osada S, Umesono K, Namura S (2003). Brain protection by resveratrol and fenofibrate against stroke requires peroxisome proliferator-activated receptor *α* in mice. *Neuroscience Letters*.

[19] Howitz KT, Bitterman KJ, Cohen HY (2003). Small molecule activators of sirtuins extend Saccharomyces cerevisiae lifespan. *Nature*.

[20] Evans RM, Barish GD, Wang Y-X (2004). PPARs and the complex journey to obesity. *Nature Medicine*.

[21] Kawada T, Goto T, Hirai S (2008). Dietary regulation of nuclear receptors in obesity-related metabolic syndrome. *Asia Pacific Journal of Clinical Nutrition*.

[22] Ordentlich P, Downes M, Evans RM (2001). Corepressors and nuclear hormone receptor function. *Current Topics in Microbiology and Immunology*.

[23] Jepsen K, Rosenfeld MG (2002). Biological roles and mechanistic actions of corepressor complexes. *Journal of Cell Science*.

[24] Privalsky ML (2004). The role of corepressors in transcriptional regulation by nuclear hormone receptors. *Annual Review of Physiology*.

[25] Lefebvre P, Chinetti G, Fruchart J-C, Staels B (2006). Sorting out the roles of PPAR *α* in energy metabolism and vascular homeostasis. *Journal of Clinical Investigation*.

[26] Wang Y-X, Lee C-H, Tiep S (2003). Peroxisome-proliferator-activated receptor delta activates fat metabolism to prevent obesity. *Cell*.

[27] Tanaka T, Yamamoto J, Iwasaki S (2003). Activation of peroxisome proliferator-activated receptor delta induces fatty acid beta-oxidation in skeletal muscle and attenuates metabolic syndrome. *Proceedings of the National Academy of Sciences of the United States of America*.

[28] Semple RK, Chatterjee VKK, O’Rahilly S (2006). PPAR gamma and human metabolic disease. *Journal of Clinical Investigation*.

[29] Vanden Berghe W, Vermeulen L, Delerive P, De Bosscher K, Staels B, Haegeman G (2003). A paradigm for gene regulation: inflammation, NF-*κ*B and PPAR. *Advances in Experimental Medicine and Biology*.

[30] Delerive P, De Bosscher K, Besnard S (1999). Peroxisome proliferator-activated receptor *α* negatively regulates the vascular inflammatory gene response by negative cross-talk with transcription factors NF-*κ*B and AP-1. *Journal of Biological Chemistry*.

[31] Lee C-H, Chawla A, Urbiztondo N, Liao D, Boisvert WA, Evans RM (2003). Transcriptional repression of atherogenic inflammation: modulation by PPARdelta. *Science*.

[32] Ricote M, Li AC, Willson TM, Kelly CJ, Glass CK (1998). The peroxisome proliferator-activated receptor-*γ* is a negative regulator of macrophage activation. *Nature*.

[33] Pascual G, Fong AL, Ogawa S (2005). A SUMOylation-dependent pathway mediates transrepression of inflammatory response genes by PPAR-gamma. *Nature*.

[34] Fernandez-Real JM, Ricart W (2003). Insulin resistance and chronic cardiovascular inflammatory syndrome. *Endocrine Reviews*.

[35] Yu Y-H, Ginsberg HN (2005). Adipocyte signaling and lipid homeostasis: sequelae of insulin-resistant adipose tissue. *Circulation Research*.

[36] Yu R, Kim C-S, Kwon B-S, Kawada T (2006). Mesenteric adipose tissue-derived monocyte chemoattractant protein-1 plays a crucial role in adipose tissue macrophage migration and activation in obese mice. *Obesity*.

[37] Suganami T, Nishida J, Ogawa Y (2005). A paracrine loop between adipocytes and macrophages aggravates inflammatory changes: role of free fatty acids and tumor necrosis factor *α*. *Arteriosclerosis, Thrombosis, and Vascular Biology*.

[38] Elson CE, Underbakke GL, Hanson P, Shrago E, Wainberg RH, Qureshi AA (1989). Impact of lemongrass oil, an essential oil, on serum cholesterol. *Lipids*.

[39] Miller AL (1998). Dimercaptosuccinic acid (DMSA), a non-toxic, water-soluble treatment for heavy metal toxicity. *Alternative Medicine Review*.

[40] Takahashi N, Kawada T, Goto T (2002). Dual action of isoprenols from herbal medicines on both PPAR*γ* and PPAR*α* in 3T3-L1 adipocytes and HepG2 hepatocytes. *FEBS Letters*.

[41] He L, Mo H, Hadisusilo S, Qureshi AA, Elson CE (1997). Isoprenoids suppress the growth of murine B16 melanomas in vitro and in vivo. *Journal of Nutrition*.

[42] Burke YD, Stark MJ, Roach SL, Sen SE, Crowell PL (1997). Inhibition of pancreatic cancer growth by the dietary isoprenoids farnesol and geraniol. *Lipids*.

[43] Yu SG, Hildebrandt LA, Elson CE (1995). Geraniol, an inhibitor of mevalonate biosynthesis, suppresses the growth of hepatomas and melanomas transplanted to rats and mice. *Journal of Nutrition*.

[44] Roullet J-B, Luft UC, Xue H (1997). Farnesol inhibits L-type Ca^2+^ channels in vascular smooth muscle cells. *Journal of Biological Chemistry*.

[45] Bradfute DL, Simoni RD (1994). Non-sterol compounds that regulate cholesterogenesis. Analogues of farnesyl pyrophosphate reduce 3-hydroxy-3-methylglutaryl-coenzyme A reductase levels. *Journal of Biological Chemistry*.

[46] Lehmann JM, Moore LB, Smith-Oliver TA, Wilkison WO, Willson TM, Kliewer SA (1995). An antidiabetic thiazolidinedione is a high affinity ligand for peroxisome proliferator-activated receptor *γ* (PPAR*γ*). *Journal of Biological Chemistry*.

[47] Staels B, Dallongeville J, Auwerx J, Schoonjans K, Leitersdorf E, Fruchart J-C (1998). Mechanism of action of fibrates on lipid and lipoprotein metabolism. *Circulation*.

[48] Swiezewska E, Danikiewicz W (2005). Polyisoprenoids: structure, biosynthesis and function. *Progress in Lipid Research*.

[49] Baxter JH (1968). Absorption of chlorophyll phytol in normal man and in patients with Refsum’s disease. *Journal of Lipid Research*.

[50] Brown PJ, Mei G, Gibberd FB (1993). Diet and Refsum’s disease. The determination of phytanic acid and phytol in certain foods and the application of this knowledge to the choice of suitable convenience foods for patients with Refsum’s disease. *Journal of Human Nutrition and Dietetics*.

[51] van den Brink DM, Wanders RJA (2006). Phytanic acid: production from phytol, its breakdown and role in human disease. *Cellular and Molecular Life Sciences*.

[52] Schluter A, Yubero P, Iglesias R, Giralt M, Villarroya F (2002). The chlorophyll-derived metabolite phytanic acid induces white adipocyte differentiation. *International Journal of Obesity and Related Metabolic Disorders*.

[53] Kitareewan S, Burka LT, Tomer KB (1996). Phytol metabolites are circulating dietary factors that activate the nuclear receptor RXR. *Molecular Biology of the Cell*.

[54] Ellinghaus P, Wolfrum C, Assmann G, Spener F, Seedorf U (1999). Phytanic acid activates the peroxisome proliferator-activated receptor *α* (PPAR*α*) in sterol carrier protein 2-/sterol carrier protein x-deficient mice. *Journal of Biological Chemistry*.

[55] Gloerich J, van Vlies N, Jansen GA (2005). A phytol-enriched diet induces changes in fatty acid metabolism in mice both via PPAR*α*-dependent and—independent pathways. *Journal of Lipid Research*.

[56] Baxter JH (1968). Absorption of chlorophyll phytol in normal man and in patients with Refsum’s disease. *Journal of Lipid Research*.

[57] Goto T, Takahashi N, Kato S (2005). Phytol directly activates peroxisome proliferator-activated receptor *α* (PPAR*α*) and regulates gene expression involved in lipid metabolism in PPAR*α*-expressing HepG2 hepatocytes. *Biochemical and Biophysical Research Communications*.

[58] Aranda FJ, Villalain J (1997). The interaction of abietic acid with phospholipid membranes. *Biochimica et Biophysica Acta*.

[59] Mitani K, Fujioka M, Uchida A, Kataoka H (2007). Analysis of abietic acid and dehydroabietic acid in food samples by in-tube solid-phase microextraction coupled with liquid chromatography-mass spectrometry. *Journal of Chromatography A*.

[60] Fernandez MA, Tornos MP, Garcia MD, de las Heras B, Villar AM, Saenz MT (2001). Anti-inflammatory activity of abietic acid, a diterpene isolated from Pimenta racemosa var. grissea. *Journal of Pharmacy and Pharmacology*.

[61] Takahashi N, Kawada T, Goto T (2003). Abietic acid activates peroxisome proliferator-activated receptor-*γ* (PPAR*γ*) in RAW264.7 macrophages and 3T3-L1 adipocytes to regulate gene expression involved in inflammation and lipid metabolism. *FEBS Letters*.

[62] Kang M-S, Hirai S, Goto T (2008). Dehydroabietic acid, a phytochemical, acts as ligand for PPARs in macrophages and adipocytes to regulate inflammation. *Biochemical and Biophysical Research Communications*.

[63] Kang M-S, Hirai S, Goto T (2009). Dehdroabietic acid,a diterpene improves diabetes and hyperlipdemia in obese diabitic KK-Ay mice. *BioFactors*.

[64] Ito C, Itoigawa M, Ju-Ichi M (2005). Antitumor-promoting activity of coumarins from citrus plants. *Planta Medica*.

[65] Montonen J, Jervinen R, Heliovaara M, Reunanen A, Aromaa A, Knekt P (2005). Food consumption and the incidence of type II diabetes mellitus. *European Journal of Clinical Nutrition*.

[66] Kuroyanagi K, Kang M-S, Goto T (2008). Citrus auraptene acts as an agonist for PPARs and enhances adiponectin production and MCP-1 reduction in 3T3-L1 adipocytes. *Biochemical and Biophysical Research Communications*.

[67] Takahashi N, Kang M-S, Kuroyanagi K (2008). Auraptene, a citrus fruit compound, regulates gene expression as a PPAR*α* agonist in HepG2 hepatocytes. *BioFactors*.

[68] Giuliano G, Rosati C, Bramley PM (2003). To dye or not to dye: biochemistry of annatto unveiled. *Trends in Biotechnology*.

[69] Bouvier F, Dogbo O, Camara B (2003). Biosynthesis of the food and cosmetic plant pigment bixin (annatto). *Science*.

[70] Kiokias S, Gordon MH (2003). Dietary supplementation with a natural carotenoid mixture decreases oxidative stress. *European Journal of Clinical Nutrition*.

[71] Reddy MK, Alexander-Lindo RL, Nair MG (2005). Relative inhibition of lipid peroxidation, cyclooxygenase enzymes, and human tumor cell proliferation by natural food colors. *Journal of Agricultural and Food Chemistry*.

[72] Silva CR, Greggi-Antunes LM, Bianchi MDLP (2001). Antioxidant action of bixin against cisplatin-induced chromosome aberrations and lipid peroxidation in rats. *Pharmacological Research*.

[73] Russell KRM, Omoruyi FO, Pascoe KO, Morrison EYA (2008). Hypoglycaemic activity of Bixa orellana extract in the dog. *Methods and Findings in Experimental and Clinical Pharmacology*.

[74] Fernandes ACS, Almeida CA, Albano F (2002). Norbixin ingestion did not induce any detectable DNA breakage in liver and kidney but caused a considerable impairment in plasma glucose levels of rats and mice. *Journal of Nutritional Biochemistry*.

[75] Takahashi N, Goto T, Taimatsu A (2009). Bixin regulates mRNA expression involved in adipogenesis and enhances insulin sensitivity in 3T3-L1 adipocytes through PPAR*γ* activation. *Biochemical and Biophysical Research Communications*.

[76] Giugliano D, Ceriello A, Esposito K (2008). Are there specific treatments for the metabolic syndrome?. *American Journal of Clinical Nutrition*.

